# Polymorphic Behavior and Phase Transition of Poly(1-Butene) and Its Copolymers

**DOI:** 10.3390/polym10050556

**Published:** 2018-05-21

**Authors:** Rui Xin, Jie Zhang, Xiaoli Sun, Huihui Li, Zhongjie Ren, Shouke Yan

**Affiliations:** 1 State Key Laboratory of Chemical Resource Engineering, Beijing University of Chemical Technology, Beijing 100029, China; xin_r@126.com (R.X.); xiaolisun@mail.buct.edu.cn (X.S.); lihuihui@mail.buct.edu.cn (H.L.); renzj@mail.buct.edu.cn (Z.R.); 2 Key Laboratory of Rubber-Plastics, Qingdao University of Science & Technology, Qingdao 266042, China

**Keywords:** poly(1-butene), polymorph, crystal structure, phase transition, kinetics

## Abstract

The properties of semicrystalline polymeric materials depend remarkably on their structures, especially for those exhibiting a polymorphic behavior. This offers an efficient way to tailor their properties through crystal engineering. For control of the crystal structure, and therefore the physical and mechanical properties, a full understanding of the polymorph selection of polymers under varied conditions is essential. This has stimulated a mass of research work on the polymorphic crystallization and related phase transformation. Considering that the isotactic poly(1-butene) (iPBu) exhibits pronounced polymorphs and complicated transition between different phases, the study on its crystallization and phase transformation has attracted considerable attention during the past decades. This review provides the context of the recent progresses made on the crystallization and phase transition behavior of iPBu. We first review the crystal structures of known crystal forms and then their formation conditions and influencing factors. In addition, the inevitable form II to form I spontaneous transition mechanism and the transformation kinetics is reviewed based on the existing research works, aiming for it to be useful for its processing in different phases and the further technical development of new methods for accelerating or even bypass its form II to form I transformation.

## 1. Introduction

Polymorphism of polymers, namely crystallization of a given polymer into different crystal structures, is aubiquitous phenomenon in material science [1,2,3,4,5,6,7,8,9,10,11,12,13,14,15,16,17,18,19]. As examples, the poly(vinylidene fluoride) exhibit five crystalline modifications [6,7,8,9], while the isotactic poly(1-butene) (iPBu) present at least four known structures [16,17]. It is well known that variation in structures can alter the physical and mechanical properties significantly. Therefore, the study on the crystallization of semicrystalline polymers in different forms under various conditions has attracted much attention over the years. The crystallization of polymer is realized by the ordered packing of molecular chain segments, which have low energy regular conformation, into a unit cell. Consequently, polymorphism of polymeric materials is generally based on the existence of several possible low-energy conformations of a given polymer. IPBu is a representative example for this type of polymorphism. The form I, II and III crystals of iPBu are constructed by the 3/1, 11/3 and 4/1 helical chains, respectively [15]. The polymorphism of polymeric materials can also arise from different packing modes of the building units possessing identical regular conformation. An example of this polymorphism typology is isotactic polypropylene (iPP). The same threefold helical iPP chain segments can pack into a monoclinic, trigonal, or orthorhombic lattices corresponding to its α, β, and γ forms, respectively [3,4].

As is well known, the stability of various crystals for a given polymer is different. There always exists a thermodynamically most stable phase among different modifications, such as form I of iPBu. Other less stable modifications are referred to as metastable phases at a specific condition [20]. The metastable phases are considered to fall into one of the multiple local free energy minima in the Gibbs free energy profile. The difference in stability of different crystals offers the possibility of phase transition between them via suitable treatment. Taking all these into account, an understanding of the formation and transformation mechanisms of different crystal modifications is of great importance for a purposeful structural control of the semicrystalline polymers.

The purpose of this review is to outline the significance of the research and development that has been undertaken towards an understanding of the formation and transformation mechanisms of iPBu related materials. After a brief description of the crystal structures related to different modifications, we focus mainly on the regulation of the crystal modification and the pathway for controlling the related phase transition of it rather than simply summarizing all of the work to date on this issue.

## 2. Polymorphic Behavior of iPBu

**Figure 1:**
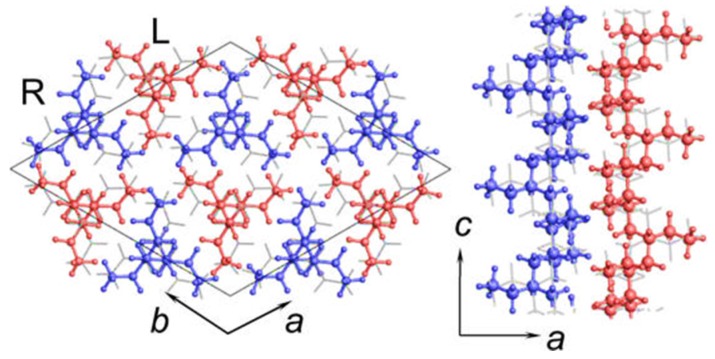
Crystal structure of iPBu form I looking along the *c*- (**left**) and *b*-axes (**right**), respectively. Reproduced with permission from Ref. [23], copyright 2016 American Chemical Society.

IPBu is one of the important multipurpose polymers. It can crystallize into four different crystal structures, designated as forms I, II, III, and I’, depending on its crystallization conditions [15,16,17,20,21,22,23,24,25,26,27,28,29,30]. Form I is composed of left- and right-handed 3/1 helix chain segments in a trigonal (hexagonal-type) crystal structure [17,21]. In this crystal structure, the right- and left-handed helical chains form pairs and pack together around the three-fold rotation axis in a way that the right-handed helixes are surrounded by the left-handed helixes and vice versa. There exist two possible space groups: *R*3*c* or $R\bar{3}c$ Based on quantitative analysis of the two-dimensional X-ray diffraction data, Tashiro and coworkers [20] suggested an $R\bar{3}c$ space group of form I in 1997. However, the same research group further refined the crystal structure of form I in a very recent study [23]. As shown in Figure 1, a $P\bar{3}$ space group is now believed to be the crystal structure of form I.

**Figure 2:**
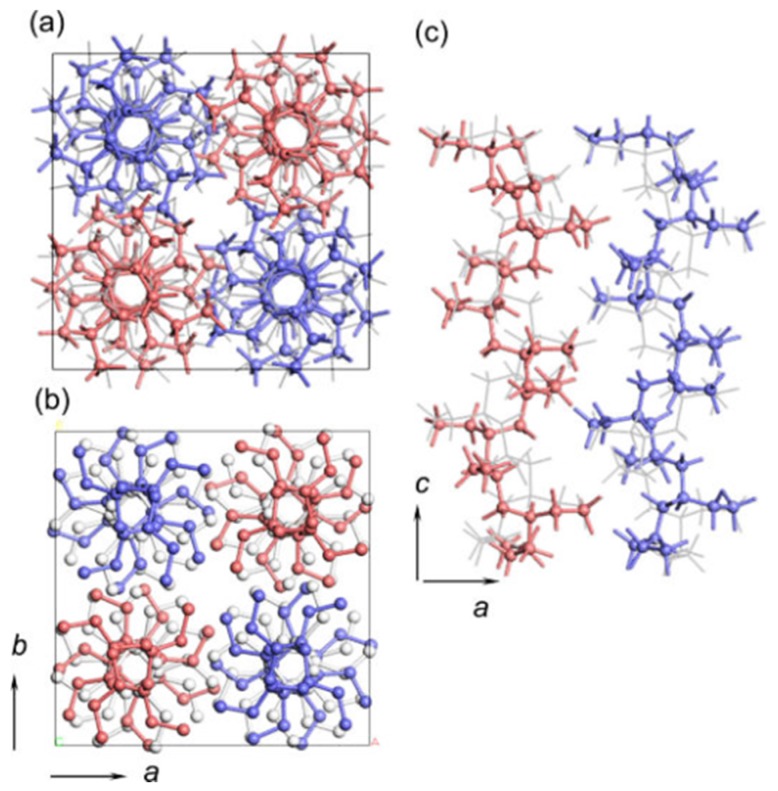
Crystal structure of iPBu form II looking along the *c*- (**a**,**b**) and *b*-axes (**c**), respectively. Reproduced with permission from Ref. [23], copyright 2016 American Chemical Society.

Form II has a tetragonal unit cell packed by 11/3 helix conformation with opposite chirality and a repeating period along the chain axis of 21 Å [24,25,26]. It is recently confirmed to have a $P\bar{4}b2$ space group. As presented in Figure 2, the right- and left-handed helical chains are positioned alternately with the statistical disorderliness about the up- and down-ward directionality [23].

Form III exhibits an orthorhombic unit cell with 4/1 helix chains [27,28]. The space group is P2_1_2_1_2_1_ with cell constants *a* = 12.38, *b* = 8.88 and *c* = 7.56 Å. The fourth polymorph, namely form I’, is actually an untwined variant of form I. It is produced in a different way as form I but shows exactly the same crystal structure with form I [29]. However, their melting temperatures have been reported to be significantly different. Since forms III and I’ can only be obtained either by crystallization from certain dilute solutions or through crystallization from melt under high hydrostatic pressure [26,30], they are lack of practical interests. Therefore, most of the studies on iPBu focus mainly on the crystallization and transformation behavior of forms I and II.

## 3. Crystal Structural Control of iPBu

The crystallization of semicrystalline polymers can be either thermodynamically controlled or kinetically favored. Whatever the crystallization mechanism, the polymer crystallization takes place generally in one of the polymorphs, either a stable one or its metastable counterpart, under certain condition. For example, while the crystallization of iPP either from the quiescent melt or from the solution generates the most stable α crystals, the crystallization of iPBu from the quiescent melt always produces the metastable form II. The formation of other crystalline phase can only be realized under specific crystallization process. In this case, the control of the crystal modification of polymorphic polymers is extremely important for design materials with required performance. This leads to the study on the crystallization behavior of polymers with pronounced polymorphs, an everlasting topic in the field of polymer physics. It is now clear that the modification selection of polymorph polymers depends on many factors, such as temperature, molecular characteristics, external fields, and the supporting or filler substances. In this section, we mainly introduce some factors that influence the selection of iPBu modification.

### 3.1. Influence of Temperature on the Polymorphism of iPBu

**Figure 3:**
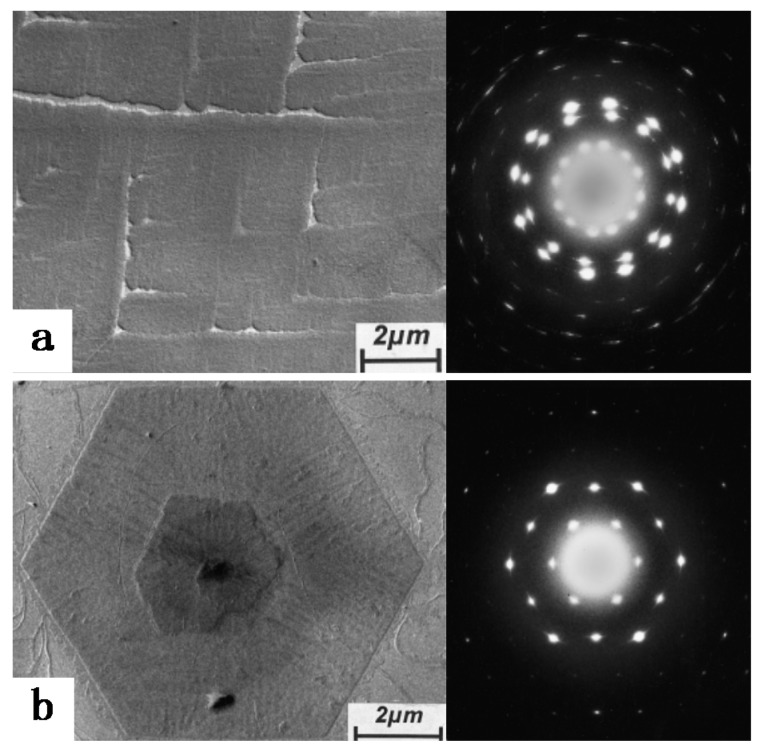
Bright field electron micrographs and corresponding diffraction patterns of iPBu form I crystals converted from form II (**a**) and crystallized directly from the melt (**b**). The sample used for (**a**) was prepared by heating the sample up to 160 °C for 15 min, then isothermally crystallized at 95 °C for 30 min, and finally aged at room temperature for three months. The sample used for (**b**) was prepared by heating the sample up to 160 °C for 15 min and then isothermally crystallized at 110 °C for fivedays. Reproduced with permission from Ref. [33], copyright 2002 Wiley Periodicals, Inc.

**Figure 4:**
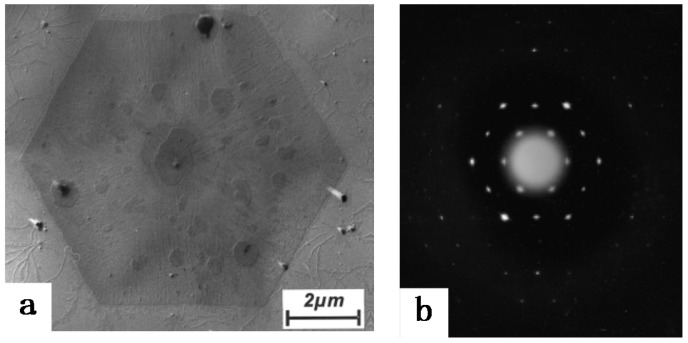
Bright field electron micrograph (**a**) and corresponding diffraction pattern (**b**) of iPBu form I crystallized directly from the melt after heating up to 125 °C for 15 min and then cooled to room temperature. Reproduced with permission from Ref. [33], copyright 2002 Wiley Periodicals, Inc.

As temperature plays a very important role universally in any crystallization process of polymers, we first describe the unique influence of temperature on the crystallization of iPBu in this part. The temperature effect on the crystallization of polymers is twofold, including the melting temperature for sample preparation as well as the temperature for subsequent crystallization. For iPBu, it is well confirmed that crystallization from the melt can only produce the metastable form II phase at atmospheric pressure and high supercooling, which converts into form I spontaneously at room temperature [31,32]. Considering that the transformation, which can take days or weeks, corresponds to a densification and shrinkage of the molded objects, bypassing the unstable tetragonal crystallization is of great significance from a practical standpoint. The direct formation of form I crystals has been achieved for ultrathin film crystallization at very low supercooling [33]. The directly formed form I and transformed form I exhibit different crystalline morphology. As presented in Figure 3, melt-grown form I iPBu single crystals have a well-shaped hexagonal form, whereas form I crystals converted from form II display the morphology of their tetragonal precursors. The electron diffraction results indicate that, instead of the twined hexagonal pattern of the converted form I crystal, the directly formed form I single crystals exhibits an untwined hexagonal pattern. The untwined diffraction feature has a close resemblance as the form I’ single crystals produced from solution and therefore may be recklessly assigned to its form I’ modification. This is actually not the case. Form I’ has a lower melting temperature of ca. 95 °C. The morphology and crystal structure of the melt-grown form I iPBu single crystals do not change by heating the sample up to 125 °C for 15 min and then cooling to room temperature (Figure 4). This clearly indicates itshigher thermal stability than the solution grown form I’ crystals and confirms the direct formation of form I from melt rather than form I’.

Besides the crystallization temperature, the melting temperature during sample preparation on the crystallization is ubiquitous for any polymer. It has been shown that the melting status exerts remarkable influence on the polymorphism of semicrystalline polymers. The effect of melting temperature on the subsequent crystallization rests on the existence of surviving crystallites or oriented segments when a polymer is melted just above its nominal melting temperature but well below the equilibrium melting temperature [34,35,36,37]. Therefore, the melting status can be divided into two cases. One is the partial melting of the crystals known as “self-seeding”, also referred frequently to as “self-nucleation”, in which residual pristine crystal fragments survive. These survived crystal fragments serve directly as crystal nuclei and influence the subsequent crystallization. In the other case, the crystal structure is completely destroyed. A full relaxation of the molecular chains is, however, not reached. There may be local clusters of aligned chain segments previously included in the crystal lattice. These aligned chain clusters can easily transform into the crystal nuclei and therefore exert important influence on the subsequent crystallization. 

**Figure 5:**
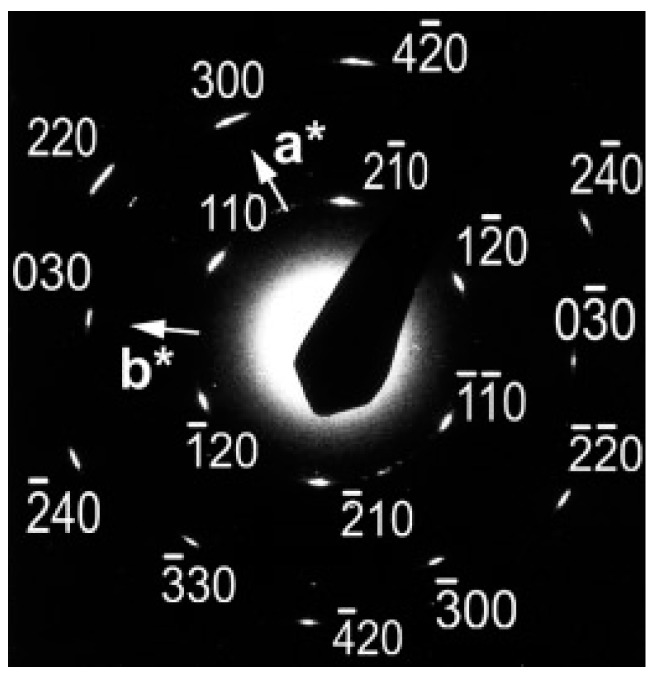
Electron diffraction pattern of an iPBu single flat-on crystal in the trigonal form. The sample was prepared by melting the solution-grown trigonal crystals at 136 °C for 2 min and then crystallized isothermally at 75 °C. Reproduced with permission from Ref. [46], copyright 2007 Wiley Periodicals, Inc.

For the first case, the partial melting is usually carried out at a selected temperature close to but below the melting temperature. In this case, the surviving crystal nuclei in the original sample will significantly affect crystallization behavior of the molten part, which can exert control over the kinetics of the process and on the re-crystallizing structure [38,39,40,41,42,43,44]. As an example, the form I iPBu crystals can only be obtained from melt crystallization at specific conditions, such as high pressure [30,45] or high temperature ultrathin film crystallization [33]. Starting from solution-grown trigonal crystals, Yamashita et al. [46] has successfully observed the growth of trigonal phase from melt via self-seeding at atmospheric pressure. They found that when the iPBu trigonal crystals were heated up to a temperature above 142 °C (well above the melting point of the trigonal phase), all of the trigonal crystals were completely melted and only iPBu tetragonal phase grown out on cooling to a crystallization temperature. On the contrary, when the samples were heated up to 136 °C for 2 min, melting of the iPBu trigonal crystals was incomplete and numbers of trigonal nuclei eventually survived from the melting. These surviving trigonal nuclei enable the trigonal crystals to grow in the melt even at atmospheric pressure and lower crystallization temperatures on cooling. The thus prepared iPBu single crystals exhibit also an untwined hexagonal electron diffraction pattern, as presented in Figure 5. In situ melting experiment shows that the melting temperature of these trigonal crystals is much higher than the melting point of form I’, indicating that they are characterized as form I trigonal crystals grown in the melt. The self-seeding induced form I crystal regrowth of iPBu has also been observed for the form I iPBu crystals converted from form II [47,48,49]. To this end, the iPBu samples were first heated up to 180 °C for 5 min to erase their previous thermos-mechanical history. After that, the molten samples were cooled to a chosen temperature for producing form II iPBu crystals and then aged at room temperature to realize the form II to form I transition. The thus obtained form I crystal can be used for the self-seeding experiments. It was confirmed that sole form I iPBu crystals can be successfully produced under elaborately selected proper self-nucleation temperature and subsequent recrystallization [50]. 

**Figure 6:**
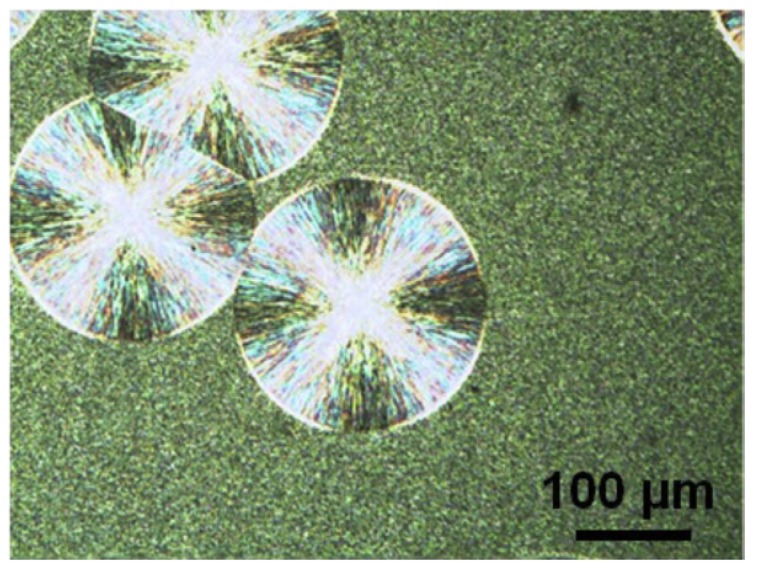
The optical micrograph showing the dual morphology of the iPBu samples prepared by crystallizing from melt first at 90 °C isothermally for 10 min and then quenching to room temperature. Reproduced with permission from Ref. [49], copyright 2013 Elsevier Ltd.

**Figure 7:**
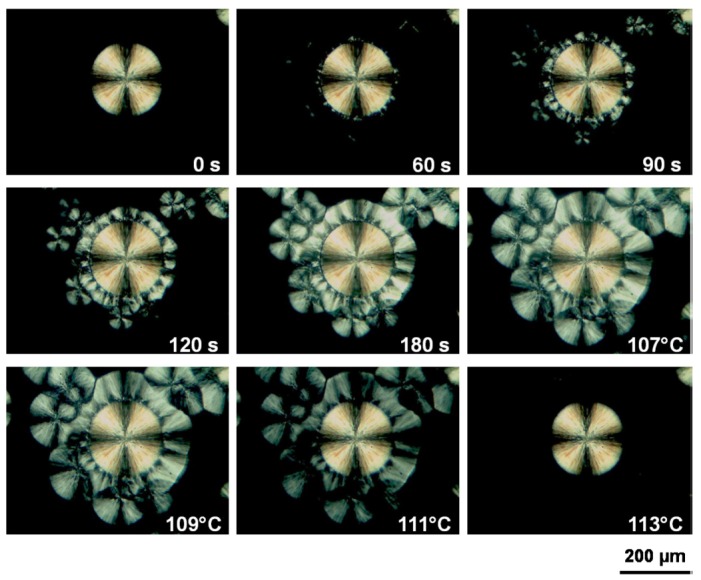
Optical micrographs showing the induced crystallization of a form I iPBu spherulite toward the surrounding molten part and the in situ melting process of the newly formed iPBu crystals. The pictures were taken during the isothermal crystallization process at 83 °C from 0 to 120 s and the in situ heating process from 83 to 113 °C. Reproduced with permission from Ref. [49], copyright 2013 Elsevier Ltd.

It should be pointed out that nucleation of one polymorph on the crystal of itself is most frequently occurred during self-seeding. However, the nucleation of another polymorph on the previously existing one also happens in some cases. The phenomenon of crystallization between polymorphs has been termed as “cross-nucleation” [51], which means a daughter phase nucleated on a pre-existing polymorph and has been previously referred to as crystal growth transformation [52,53,54,55,56]. It has received much attention recently [57,58,59,60,61]. Cavallo and coworkers have performed systematic studies on cross-nucleation of iPBu [49,50,62,63]. In their work, a dual crystalline morphology of iPBu is elaborately produced by crystallizing first at 90 °C isothermally for a short time and then quenching to room temperature. Figure 6 presents the typical morphology of thus prepared sample. It contains a small number of large spherulites crystallized at 90 °C and abundant microcrystallites generated during quenching. The sample shown in Figure 6 was subsequently aged at room temperature for four weeks to ensure a completely form II to form I solid phase transformation. Afterwards, it has been heated up to 120 °C for melting the surrounding microcrystallites crystallized at room temperature but keeping the isothermally grown big spherulites intact. In this way, the induced-crystallization of the molten part by the existing form I iPBu crystals has been followed by cooling down to different temperatures. Figure 7 is the optical micrographs showing the induced crystallization of a form I iPBu spherulite toward the molten part at 83 °C and the subsequent melting process of the newly formed iPBu crystals. It can be clearly seen that a big spherulite is indeed survived during melting at 120 °C. By cooling down from 120 to 83 °C again, see Figure 7 at 0 s, the big spherulite remains unchanged, whereas the recrystallization of the surrounding melt does not take place. After 60 s holding at 83 °C, recrystallization of the molten iPBu starts both in the amorphous bulk and on the periphery of the big spherulite. In addition, all of the newly formed crystals molten away at 113 °C in the reheating process. This demonstrates unambiguously that the recrystallized crystals are form II of iPBu, indicating the occurrence of cross-nucleation.

**Figure 8:**
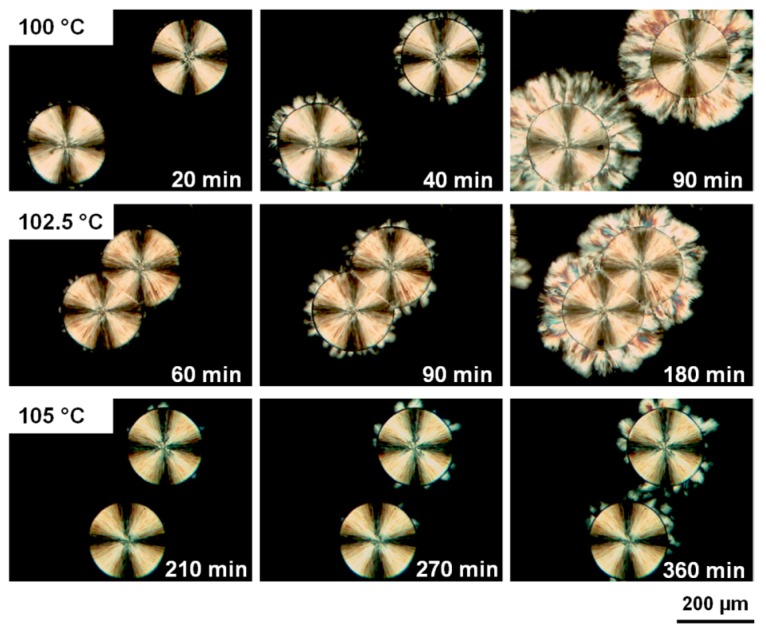
Optical micrographs taken during the form I induced form II crystallization of iPBu at different temperatures and for times as indicated in the pictures. Reproduced with permission from Ref. [62], copyright 2014 American Chemical Society.

Moreover, it can be recognized in Figure 7 that the nucleation at the periphery of the previous spherulite is predominant compared with that in the bulk. This has been more clearly identified in the temperature dependent crystallization process, as presented in Figure 8. For example, when the crystallization temperature raises from 83 to 100 °C (top panel of Figure 8), while the form I iPBu spherulite has been completely encompassed after ca. 40 min isothermal crystallization, the crystallization of the remaining bulk does even not start. This implies that the form I seeds can more efficiently nucleate their form II counterpart than commonly available sources of heterogeneous nuclei in the iPBu melt. Of course, the cross-nucleation events become progressively rarer with increasing temperature. In other words, the cross-nucleation frequency of form II on form I decreases at higher temperatures. Taking the crystallization at 105 °C (bottom panel of Figure 8) as an example, the nucleation and crystallization of form II iPBu can only be observed at several places on the boundary of the form I spherulite. 

**Figure 9:**
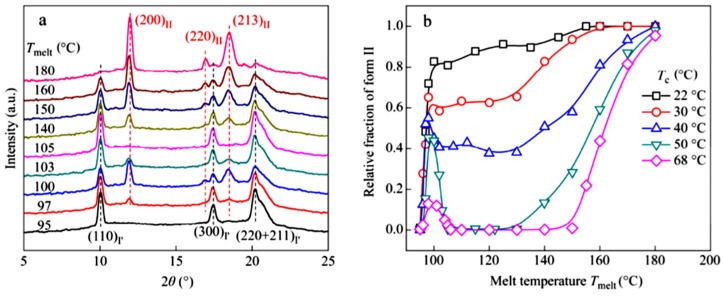
(**a**) Selected WAXD curves of a butene-1/ethylene random copolymer with 9.88 mol % ethylene co-unit crystallized isothermally at 50 °C after melting at different temperatures as indicated in the left column. (**b**) The relationship between the relative amount of form II crystals crystallized at different temperatures as indicated in the right column and the melting temperature during sample preparation. Reproduced with permission from Ref. [74], copyright 2016 Chinese Chemical Society.

For the second case, when melting the sample at temperatures slightly higher than that needed for self-seeding, even though no pristine crystal fragments survived, there are local domains of aligned chain segments previously included in the crystal lattice as long as an equilibrium melting state is still not reached [64]. This originates from the long chain character of polymers, which results in a temperature-dependent relaxation of the long molecular chains. The entities of aligned chain clusters can easily transform to active nuclei during cooling and influence the subsequent crystallization behavior remarkably, which is generally referred to as the “memory effect”. It can accelerate the crystallization rate, alter the crystalline morphology and influence the selection of polymorph due to the change of free energy for nucleation [65,66,67]. For example, oriented α-iPP fiber can melt-recrystallize into its α or β phase depending on the melting status and recrystallization temperature [68,69,70,71,72]. Men and co-workers have performed detailed studies about the influence of melting status and temperature of subsequent crystallization on the phase selection of an iPBu copolymer [73,74]. It was found that form II has always been obtained after melting at high temperature (e.g., 180 °C) and then crystallized at temperatures lower than 70 °C, independent of the initial crystalline phase. This reflects a complete relaxation of the molecular chains at 180 °C. Consequently, the melt can only crystallize into its form II as generally observed for the crystallization of iPBu homopolymer. The influence of melting temperature on the subsequent crystallization behavior is well illustrated by melting the form I’ crystals at different temperatures before isothermal recrystallization at 50 °C. The ultimate melting temperature of the used form I’ crystals is estimated to be ca. 95 °C. As shown in Figure 9a, the form I’ or II crystallization depends strongly on the melting status. When melting the sample at 95 °C, only form I’ iPBu crystals were obtained. This can be related to the self-seeding process that the residue crystallites acted as nuclei leading to the recrystallization in the same crystalline phase. When melting the sample at 97 °C, besides the characteristic diffraction peaks of form I’ crystals, diffraction peaks related to the form II crystals also appear, indicating the concomitant crystallization of both forms I’ and II. The content of form II crystals increases with further increment of melting temperature to 100 °C. It should be mentioned that form II disappears again when the melting temperature set at 105 °C. Afterwards, the intensity of the form II diffraction peaks increases again with temperature until fully crystallization in form II when melted at 180 °C. 

The above-observed complex dependence of polymorph selection on the melting temperature is rather peculiar. For a better understanding of it, they further investigated the effect of crystallization temperature on the polymorph selection of the iPBu copolymer under controlled melting condition. Figure 9b shows the relative amount of form II crystals after melting the starting samples with form I’ crystals for 10 min at different *T*_melt_ and then recrystallization at the indicated crystallization temperature. It can be seen that only form I’ crystals were re-generated at any crystallization temperature when the initial form I crystals were melted at *T*_melt_ = 95 °C, reflecting the self-seeding process caused by residue crystallites. Undoubtedly, the melting extent and the relaxation level of the molecular chains increases with *T*_melt_. As a result, the influence of crystallization temperature on the polymorph selection becomes evident. It can be clearly seen in Figure 9b that, at low crystallization temperature, e.g., below 30 °C, the relative amount of form II crystals monotonically increases first very quickly with *T*_melt_ below 100 °C and then slowly with *T*_melt_ over 100 °C. However, interesting dependence of the form II fraction on the *T*_melt_ was observed when the crystallization temperature was set higher than 30 °C. For example, when crystallizing the sample at 40 °C after melting at different temperatures, the amount of form II crystals increases sharply with *T*_melt_ to 100 °C and then reduces to some extent with further increment of the *T*_melt_ to ca. 105 °C. After that, the relative amount of form II crystals increases again monotonically. If the crystallization temperature is higher than 50 °C, after a quick increase in the amount of form II crystal with *T*_melt_, the crystallization of form II has been suppressed in a *T*_melt_ window from 108 to approximately 130 °C. Afterwards, it increases again monotonically until the formation of only form II crystals. These results reveal the synergistic effect of melting and crystallization temperatures on the formation of a given polymorphic phase of iPBu. It has been explained in terms of the interplay between the domain size of aligned chain segregates previously included in the crystal lattice and the size and energy barrier of the critical nucleus corresponding to different crystalline forms. Turner-Jones [24] reported that the energy barrier for form II crystal nucleation is much lower than the one for form I’ crystals. Therefore, iPBu crystallizes preferentially in its form II from a homogeneous melt. It should be kept in mind that formation of nucleus with a critical size is the prerequisite for crystal growth. For a given polymer of certain crystal phase, the needed critical nucleus size decreases with supercooling. In other words, the higher the supercooling, the smaller the critical nucleus. In the present case, the existence of aligned chain clusters in the heterogeneous melt is another key fact that promotes the nucleation and accelerates the crystallization. As long as the domain size of aligned chain clusters is larger than critical nucleus size, the ordered chain clusters will easily transform into active nuclei that initiate the growth of crystals. Taking all these into account, an understanding on the aforementioned experimental results may be reached. At lower crystallization temperatures, e.g., the size of critical nucleus that required for iPBu crystal growth of both forms I’ and II is smaller than the domain size of aligned chain clusters. Consequently, the lower energy barrier of form II iPBu leads to a crystallization of it in form II. The required size of critical nucleus for both kinds of iPBu crystals increases with increasing crystallization temperature, while the domain size of aligned chain clusters decreases with increasing melting temperature. Moreover, the size of critical nucleus required for iPBu crystal growth of form I’ is assumed smaller than that of form II [22]. In this case, the ordered domain size in samples melted in temperature range from 108 to approximately 130 °C may larger than the critical nucleus size of form I’ but smaller than that of form II at around 50 °C. As a result, crystallization in form I’ is preferred. With further increase in melting temperature, e.g., 150 °C, there might only be few ordered domains with size over the critical nucleus size of form I’. These ordered domains enable the partial crystallization of melt into form I’ iPBu crystals. On the other hand, the lower energy barrier of form II iPBu causes the crystallization of the rest melt in form II. 

### 3.2. Influence of Molecular Characteristics on the Polymorphism of iPBu

**Figure 10:**
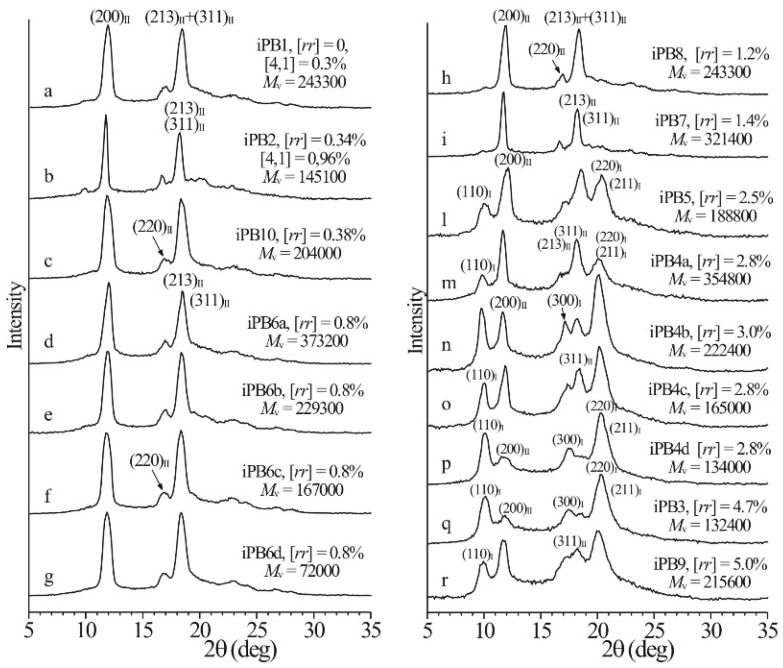
X-ray powder diffraction profiles of samples of iPBu of different stereoregularity crystallized from the melt by cooling the melt to room temperature (or lower temperature) at cooling rate of 10 °C/min. The (110)_I_, (300)_I_ and (220)_I_/(211)_I_ reflections of form I at 2θ = 9.9°, 17.3° and 20.5°, and the (200)_II_, (220)_II_ and (213)_II_/(311)_II_ reflections of form II at 2θ = 11.9°, 16.9° and 18.3° are indicated. Reproduced with permission from Ref. [80], copyright 2009 American Chemical Society.

The molecular parameters, such as molecular weight, chain architecture, stereoregularity, etc., also exhibit great influence on the polymorphism selection [10,13,14,75,76,77,78,79]. De Rosa et al. [80,81,82] have studied the effect of the molecular weight, stereo- and regiodefects on the polymorphic behavior of melt-crystallized iPB. It was found that the more stereoregular samples with concentration of [*rr*] stereodefects lower than 1.4 mol %always crystallize into form II structure from the melt regardless of molecular weight (see the X-ray diffraction profiles a–i in Figure 10). On the contrary, the iPB samples with more stereoirregular, e.g., over 2.5 mol % [*rr*] defects, crystallize from the melt as mixtures of form II and form I (it has been referred to as form I’ in Ref. [80]). In the X-ray diffraction profiles l–r in Figure 10, the content of form I increases with increasing concentration of the defects. This illustrates clearly the influence of stereoirregularity on the polymorph selection of iPBu. Moreover, the [4,1] region-defects affect the polymorphic behavior as well. It should be mentioned here that incorporation of other co-units, i.e., the copolymers, has also great effect on the polymorphic crystallization of iPBu. The influence of few co-units has a close resemblance to the stereo- and regiodefects. If the co-components is long enough for crystallization, then the co-components exhibits a mutual influence on the polymorphic crystallization of each respective phase [73,74,83,84]. Actually, besides the copolymerization, even blending the iPBu with another miscible polymer shows influence on its polymorphic crystallization [85]. 

**Figure 11:**
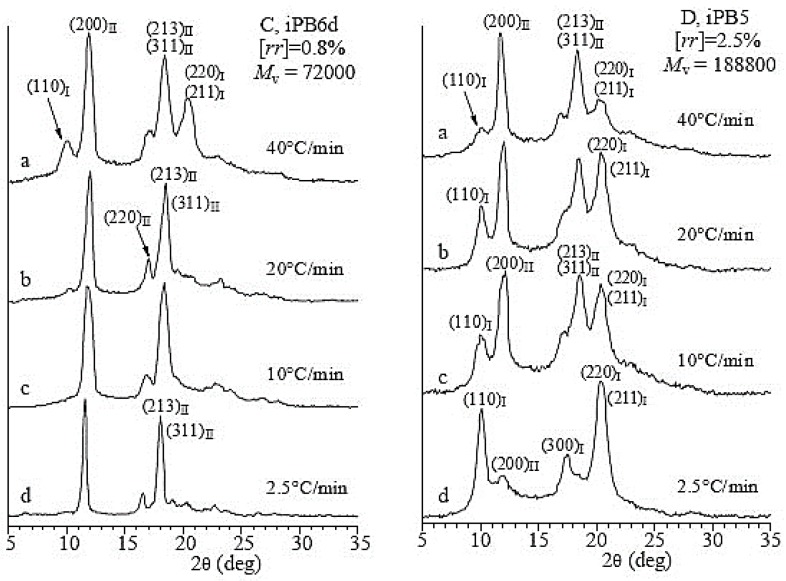
X-ray powder diffraction profiles of samples of iPBu of different stereoregularity crystallized from the melt by cooling the melt to room temperature (or lower temperature) at the indicated cooling rates. The (110)_I_, (300)_I_ and (220)_I_/(211)_I_ reflections of form I at 2θ = 9.9°, 17.3°, and 20.5°, and the (200)_II_, (220)_II_ and (213)_II_/(311)_II_ reflections of form II at 2θ = 11.9°, 16.9°, and 18.3° are indicated. Reproduced with permission from Ref. [80], copyright 2009 American Chemical Society.

**Figure 12:**
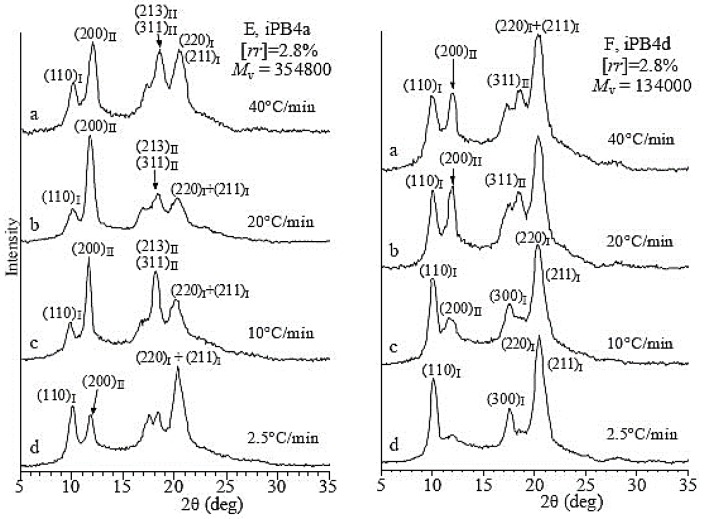
X-ray powder diffraction profiles of samples of iPBu of same stereoregularity but different molecular weight crystallized from the melt by cooling the melt to room temperature (or lower temperature) at the indicated cooling rates. The (110)_I_, (300)_I_ and (220)_I_/(211)_I_ reflections of form I at 2θ = 9.9°, 17.3° and 20.5°, and the (200)_II_, (220)_II_ and (213)_II_/(311)_II_ reflections of form II at 2θ = 11.9°, 16.9° and 18.3° are indicated. Reproduced with permission from Ref. [80], copyright 2009 American Chemical Society.

As mentioned earlier, the crystallization temperature plays a very important role universally in any crystallization process of polymers. There is no exception of the crystallization of iPBu samples with stereo- and regiodefects. Figure 11 shows the X-ray diffraction profiles of the iPBu samples with [*rr*] defects of (a) 0.8 mol % and (b) 2.5 mol % prepared by cooling the melt at varied cooling rates. For the sample with 0.8 mol % [*rr*] defects, it can be seen from Figure 11a that a fast cooling favors the crystallization of it in form I. For example, while pure form II is created by cooling the melt at a rate slower than 20 °C/min, a mixture of forms I and II can be identified when the melt was cooled at rate of 40 °C/min. For the cooling rate effect, the sample with high [*rr*] defects exhibits an opposite trend. It is clear that the content of form I in the sample with 2.5 mol % [*rr*] defects increases with decreasing cooling rate (see Figure 11b). A mixture of forms I and II is, however, always obtained for this sample under used crystallization conditions. A fully form I crystallization has successfully been realized for the sample with 5 mol % [*rr*] defects when cooling from the melt at a rate of 2.5 °C/min (see Figure 11c). For the influence of molecular weight on the polymorphic behavior of iPBu is best illustrated in Figure 12. One can see from Figure 12 that, for the samples with the same 2.8 mol % [*rr*] defects and under the same cooling rate, a lower molecular weight is more conducive to the formation of form I. The observed phenomena about the influence of stereo and regiodefects, molecular weight as well as cooling rate on the polymorphic crystallization of iPBu was explained by the balance between thermodynamic and kinetic effects.

### 3.3. Influence of External Fields on the Polymorphism of iPBu

**Figure 13:**
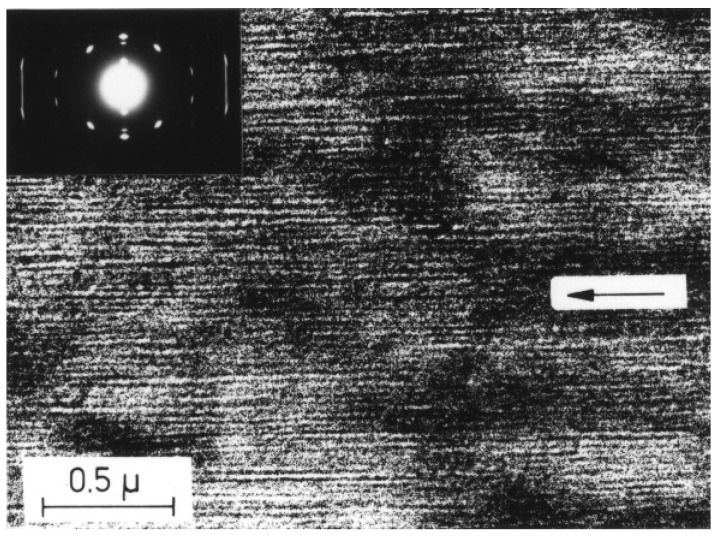
Phase contrast bright field electron micrograph and corresponding electro diffraction pattern (inset) of a melt-drawn iPBu ultrathin film recorded directly after sample preparation. The arrow indicates the drawing direction during film preparation. Reproduced with permission from Ref. [90], copyright 2003 American Chemical Society.

Crystallization in an external field is another crucial factor that exerts prominent influence on the polymorphic behavior of polymers. Elongation and flow fields are the most frequently encountered external fields for polymers during melt processing and exhibit evident influence on the crystallization of polymers [86,87]. It was well demonstrated that the iPBu exhibits form I crystal structure immediately after produced in elongation field. A most striking example for this issue is the iPBu ultrathin film produced by a melt-draw technique of Petermann and Gohil [88,89]. The melt-draw process provides a condition for high deformation within a short distance of ca. 1 μm and yields an extremely high longitudinal flow gradient of around 4 × 10^4^ s^−1^. Figure 13 shows a representative phase contrast bright field electron micrograph and corresponding electron diffraction pattern of a melt-drawn iPBu ultrathin film recorded directly after preparation. In the bright field image, needlelike crystals with a distribution of their length from 0.1 to 1 μm and a width of ca. 10 nm can be observed. The needlelike crystals aligned well along the drawing direction during film preparation, indicating a high degree of orientation. The corresponding electron diffraction pattern shows sharp and well-defined reflection spots and confirms an ordered arrangement of the iPBu crystals with molecular chains aligned in the drawing direction. All of the reflection spots have been accounted for by a trigonal unit cell with lattice parameters of *a* = *b* = 17.53 Å, *c* (fiber axis) = 6.477 Å, and γ = 120° [21]. This demonstrates that melt-drawn crystalizes directly in form I structure rather than form II as the relaxed melt does. It is reasonable since the molecular chains in the form I crystals are more extended than in form II. 

Another reported factor with distinct influence on the polymorph selection of iPBu is pressure [30,45]. It was found that, when crystallizing the iPBu at pressures below 1000 atm, form II crystals are the only products at any crystallization temperature. However, iPBu melt crystallizes directly into form I’ at pressures above 1000 atm. Crystallization at 1000 atm results in the formation of both form II and form I’ in the same sample. The proportion of form I’ iPBu increases with crystallization temperature. While the sample crystallized under 1000 atm at 85 °C contains less than 10% form I’ crystals, almost pure form I’ is obtained when crystallized at 115 °C. It was further found that crystallization under high pressure at fixed supercooling slows down the crystallization rate of form II, but does not affect the crystallization rate of form I’. 

**Figure 14:**
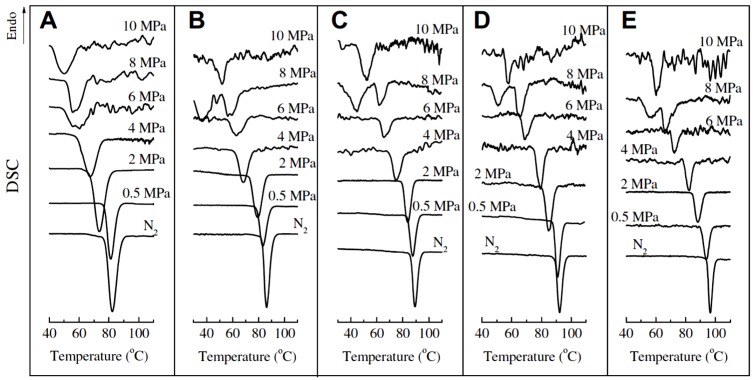
DSC diagrams recorded during non-isothermal melt-crystallization processes of iPBu under atmospheric N_2_ and CO_2_ at various pressures. The cooling rates are: (**A**) 5 °C/min; (**B**) 2.5 °C/min; (**C**) 1 °C/min; (**D**) 0.5 °C/min; and (**E**) 0.25 °C/min. Reproduced with permission from Ref. [91], copyright 2011 Elsevier Ltd.

**Figure 15:**
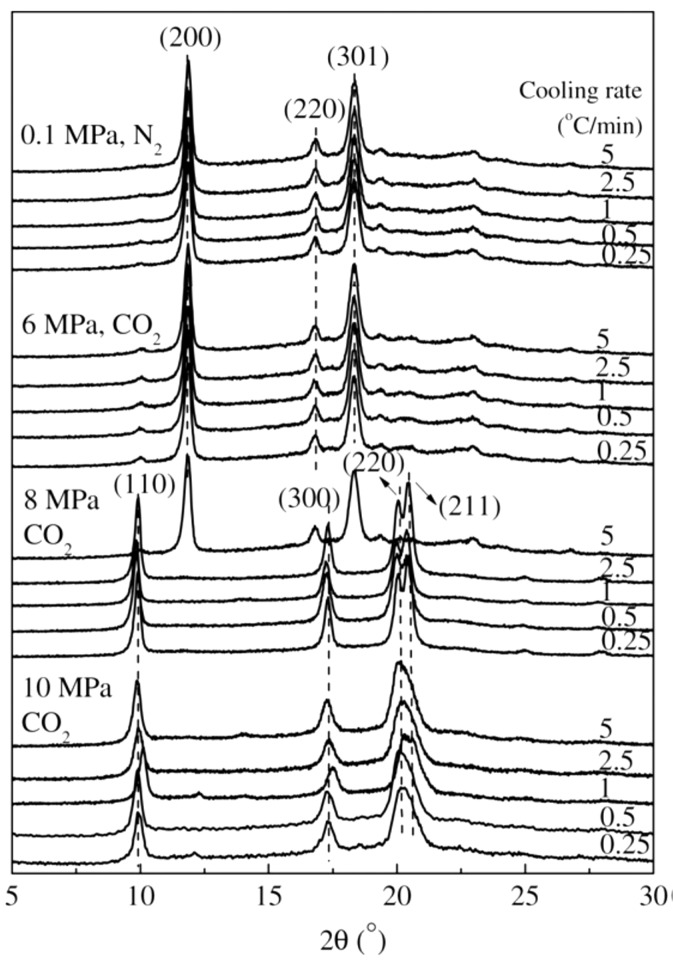
WAXD profiles of the iPBu melt-crystallized during cooling at different rates under varied conditions. Reproduced with permission from Ref. [91], copyright 2011 Elsevier Ltd.

In addition, the combination of pressure and CO_2_ makes the influence of pressure on the polymorphic behavior even outstanding. Li et al. [91,92] studied the non-isothermal melt-crystallization behavior of iPBu in compressed CO_2_ or supercritical CO_2_ under different pressures and at varied cooling rates. The DSC curves recorded during the non-isothermal crystallization are presented in Figure 14. It can be seen in Figure 14 that crystallizing the iPBu in compressed CO_2_ lowers the crystallization temperature compared with that crystallized under atmospheric N_2_. Both the onset and peak crystallization temperatures decrease steadily with increasing pressure, regardless of cooling rate. This does not mean that the cooling rate has no effect on the crystallization of iPBu in compressed CO_2_. The crystallization temperatures drop actually with increasing cooling rate at fixed pressure. Moreover, while only one exothermal peak is observed under any used pressures when cooled at a rate of 5 °C/min, two exothermal peaks appear on the DSC curves of iPBu crystallized in 8 MPa CO_2_ at cooling rates lower than 2.5 °C/min. This reveals the synergistic effects of pressure and cooling rate on the non-isothermal crystallization of iPBu in compressed CO_2_. The selected X-ray diffraction profiles corresponding to the samples crystallized under different conditions are shown in Figure 15. It was confirmed that the samples obtained in compressed CO_2_ at any cooling rate under pressures no more than 6 MPa exhibit evidently the form II crystal structure, just like that crystallized under atmospheric N_2_ (see the uppermost X-ray profile in Figure 15). The sample crystallized under 8 MPa CO_2_ shows, however, a cooling rate dependent polymorphic behavior. At a cooling rate of 5 °C/min, the formation of form II iPBu is still preferred. At all other used cooling rates, the form I iPBu crystals are obtained. For the sample crystallized under 10 MPa CO_2_, the formation of form I is not cooling rate dependent. According to the above-obtained experimental results, two things should be addressed here. First, the same crystal structure revealed by X-ray diffraction but different exotherms of iPBu crystallized under 8 and 10 MPa CO_2_ should be discussed. This is related to the different crystallization mechanisms as deduced by their melting experiments. It was confirmed that the form II iPBu crystals shows only one melting peak at ca. 119 °C, in agreement with that reported in other literatures. The form I crystals obtained under 8 MPa melt at 129 °C indicate the formation of most stable form I phase. On the contrary, the form I crystals formed under 10 MPa exhibit two melting peaks at approximately 96 and 119 °C, respectively. According to these results, it has been concluded that crystallization of iPBu under 8 MPa at cooling rate no larger than 2.5 °C/min produces initially form II crystals, which transform quickly into form I under pressure. On the other hand, Form I’ iPBu crystals form directly under 10 MPa at any cooling rates. The form I’ crystals transform into form II rather than form I during the heating process of DSC measurement. The second one rests on the different influence of pressure on the polymorphic behavior of iPBu with and without CO_2_, i.e., the growth of form II at pressures below 100 MPa without CO_2_, while of form I’ at 10 MPa with CO_2_. This has been ascribed to the plasticization effect of CO_2_ on the iPBu as also observed for other polymers [93,94].

### 3.4. Influence of Heterogeneous Entities on the Polymorphism of iPBu

Even though the aforementioned factors exhibit obvious influence on the crystallization and polymorph behavior of iPBu, the introduction of selective nucleation agents is most widely used method for preparation of samples of required crystal modification through heterogeneous nucleation [27,95,96,97,98,99,100,101,102,103,104]. The heterogeneous nucleation mechanism can be different depending on the interacting system and crystallization conditions. While Thomason et al. [105] suggested a stress-induced heterogeneous nucleation of the fiber reinforced polymer composites, Wang et al. [106] emphasized the importance of surface topography of the additives on the heterogeneous nucleation of polymers. Favored crystallographic interaction is another well-known mechanism of polymer heterogeneous nucleation. The epitaxial interactions are generally realized by matching in the crystallographic geometry, such as the coincidence of unit cell dimensions or molecular distances in the contact lattice planes based on the similarity of crystal structures between the substrate and overgrowth polymers. In this case, a subtle structural difference of the used substrates may induce completely different epitaxial crystallization of polymers. Therefore, epitaxial crystallization provides an efficient way for controlling the multistructure of polymers, such as crystal modification, crystal orientation, and even the selection of handedness of chiral polymers [107,108,109,110,111,112,113,114,115,116]. A most illustrative example is the crystallization of poly(butylene adipate) (PBA) on oriented iPP and polyethylene (PE) substrates [115,116]. On both substrates, the PBA always crystallizes in its β phase independent of crystallization temperature but with different chain orientation. A parallel chain alignment of PBA and PE is the outcome of PBA/PE epitaxy [116], whereas a crosshatched chain arrangement of PBA on iPP substrate is observed with the molecular chains ±50° apart from each other [115]. For iPBu, there is no exception. As summarized in Table 1, all three crystalline forms, including forms I’, II and III, have been successfully produced by epitaxial crystallization on appropriate low molecular weight or polymeric substrates [117,118,119,120,121]. 

**Figure 16:**
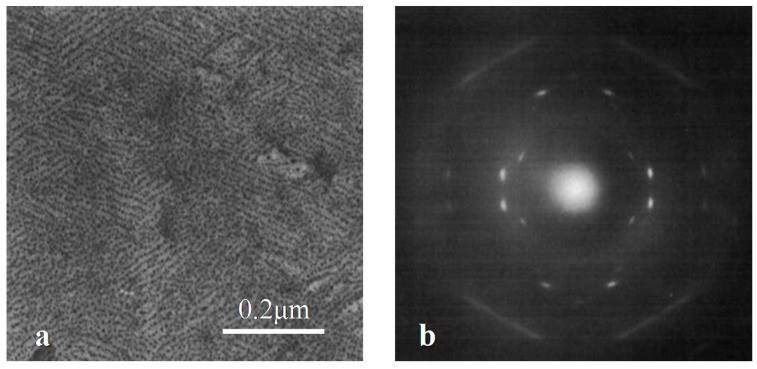
A phase contrast bright field electron micrograph (**a**) and its corresponding electron diffraction pattern (**b**) of iPBu melt-crystallized on potassium salts. Reproduced with permission from Ref. [118], copyright 1994 Butterworth-Heinemann Ltd.

**Figure 17:**
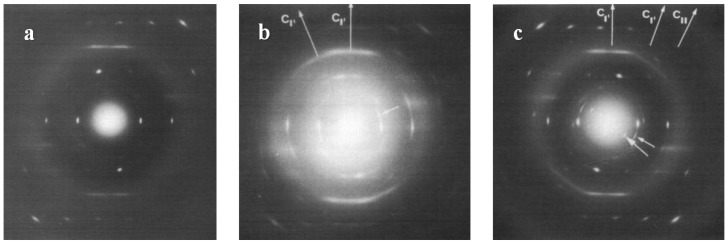
Electron diffraction patterns of iPBu melt-crystallized on 4-chlorobenzoic acid. (**a**) A single set asymmetric electron diffraction pattern of iPBu; (**b**) Two sets of form I’ crystals with molecular chains oriented 22° apart; note the difference in intensity of corresponding reflections for the two chain orientations, indicating a major and a minor component; (**c**) Two sets of form I’ crystals with chain orientations 22° apart and one set of form II chain orientation at 11° and 33° from the minor and major chain directions, respectively. Reproduced with permission from Ref. [118], copyright 1994 Butterworth-Heinemann Ltd.

It was found that purely form I’ iPBu crystals with exactly the same mutual orientation relationships could be obtained by epitaxial crystallization on potassium hydrogen 4-chlorobenzoate, potassium 4-chlorobenzoate and potassium 4-bromobenzoate substrates. Figure 16 shows a representative phase contrast bright field electron micrograph and a corresponding diffraction pattern of iPBu melt-crystallized on the aforementioned potassium salts. The appearance of crosshatched edge-on lamellar structure in the bright field image (Figure 16a) suggests a preferred two directions of chain orientation. The electron diffraction pattern (Figure 16b) consists of two set of diffraction spots corresponding to the form I’ iPBu with molecular chains 60° apart from each other. A detailed analysis of the obtained electron diffraction pattern demonstrates that the (110)_iPBu_ lattice plane is in contact with the substrate. The alignment of iPBu molecular chains in form I’ along the directions ±30° inclined to the *b*-axis of the substrate crystals leads to the formation of crosshatched lamellar structure with molecular chains of iPBu 60° apart. On the other hand, when crystallizing the iPBu from melt on 4-chlorobenzoic acid, complicated phase and orientation structures were obtained [115]. As shown in Figure 17a, a single set electron diffraction pattern of form I’ iPBu crystals is the most frequently observed. While the contact plane of iPBu is also the (110) lattice plane, the iPBu molecules aligned now along the directions ±11° away from the *c*-axis of the 4-chlorobenzoic acid substrate crystals. One can clearly see that the diffraction on the layer lines is asymmetric. This kind of asymmetry is caused by chain chirality. The asymmetric diffraction pattern shown in Figure 17a corresponds actually to exposed right-handed helices. An electron diffraction pattern corresponding to the exposed left-handed helices can be seen in Figure 17c as the main reflection set of form I’. It is in mirror symmetry with Figure 17a. In some cases, besides the main diffraction set of form I’ iPBu as that shown in Figure 17a, there appears another weak diffraction set of form I’ iPBu with its helix axis tilted 22° and its structure related by mirror symmetry to the major component in form I’. It has been associated to the tilts in opposite directions for of the left- and right-handed helices. Figure 17c shows another mixed electron diffraction pattern of iPBu crystallized on 4-chlorobenzoic acid. In this electron diffraction pattern, except for the two form I’ orientations at 22° to each other with one of them predominant, there exists also a reflection set of form II iPBu. It is caused by an epitaxial crystallization of iPBu with 11/3 helixes in (100) lattice plane along the [032] direction of the substrate also the (100) lattice plane. It should be noted here that a subtle difference in structure of the substrate can results in different epitaxy behavior of the overgrowth polymer. This has been well illustrated by the epitaxial crystallization of iPBu on 4-bromobenzoic acid. In this case, form II iPBu becomes the majority and two sets of crystal orientations with molecular chains 50° apart are observed for form II iPBu. It rests on the alignment of 11/3 helixes along the [032] and [03$\bar{2}$. ] of the substrate, respectively. However, the two form I’ orientations with 22° inclination angle are also observed, even though as a minority.

**Figure 18:**
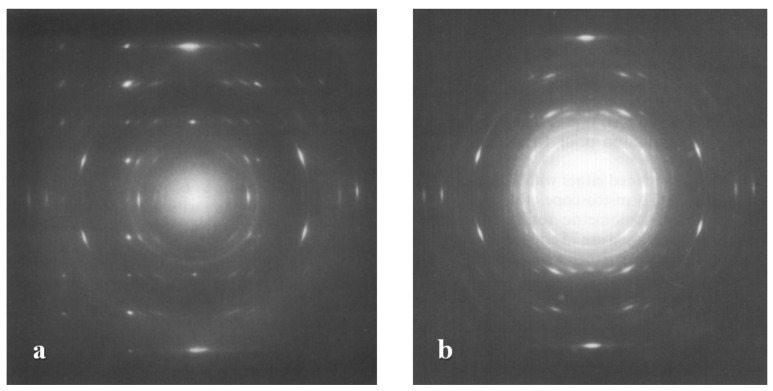
Electron diffraction patterns of iPBu crystallized on 2-Quinoxalinol substrate before (**a**) and after (**b**) removing the substrate crystals. The sharp and arced spots correspond to the diffraction by the substrate and polymer, respectively. The appearance of strong meridional spot on the fourth layer line is an indication of a 4/1 helical conformation, i.e., the formation of form III iPBu crystals. Reproduced with permission from Ref. [117], copyright 1994 Butterworth-Heinemann Ltd.

**Figure 19:**
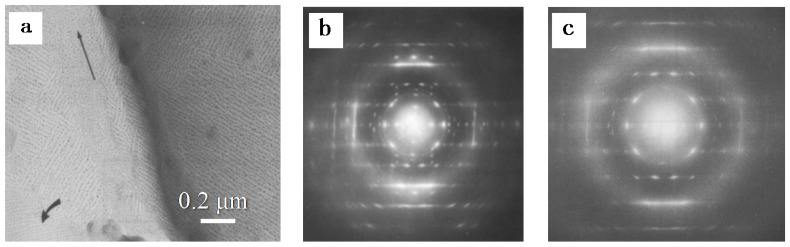
Phase contrast bright field electron micrograph (**a**) and corresponding electron diffraction patterns before (**b**) and after (**c**) the complete II to I phase transition of iPBu crystallized on benzoic acid substrate. The straight arrow in part (**a**) indicates the *b*-axis direction of the benzoic acid substrate crystals. The transformation is found to be accelerated by the electron bombardment. Reproduced with permission from Ref. [117], copyright 1994 Butterworth-Heinemann Ltd.

Form III of iPBu has been obtained by epitaxial crystallization from melt on 2-Quinoxalinol (2-Qol) [117]. Figure 18 shows the electron diffraction patterns of iPBu crystallized on 2-Qol substrate before and after removing the substrate crystals. Before dissolving the 2-Qol, the diffraction pattern is composed of superimposed diffractions of both iPBu and 2-Qol. As presented in Figure 18a, the 2-Qol crystals contribute the sharp and well-defined diffraction spots, whereas the arced reflection spots come from the iPBu crystals. This has been confirmed by dissolving the 2-Qol crystals, as displayed in Figure 18b. The location of the iPBu and 2-Qol diffraction spots on the same layer lines reveals a good match between them. Moreover, the appearance of strong meridional spot on the fourth layer line indicates a 4/1 helical conformation, i.e., the formation of form III iPBu crystals. The arrangement of (004)_iPBu_ in the same direction as the (020)_2-Qol_ demonstrates an parallel alignment of iPBu molecular chain along the *b*-axis direction of 2-Qol. When crystallizing on the on benzoic acid substrate, pure form II of the iPBu formed as in usual bulk crystallization, the form II crystals oriented, however, in a peculiar way. As displayed in Figure 19a, two populations of lamellae oriented almost perpendicularly, leading to the formation of a crosshatched lamellar structure. The corresponding electron diffraction (Figure 19b) confirms the existence of two orientations with chains 94° apart. It should be pointed out that, in the diffraction pattern of Figure 19b, some of the form II iPBu crystals have already transformed into its form I counterparts. The transformation does not change the chain orientation at all, as can be judged from the fully transformed electron diffraction pattern shown in Figure 19c. All of these examples certify the efficiency of epitaxy on the control of multistructures of iPBu. 

## 4. Phase Transition of Polymorphic Polymers

**Figure 20:**
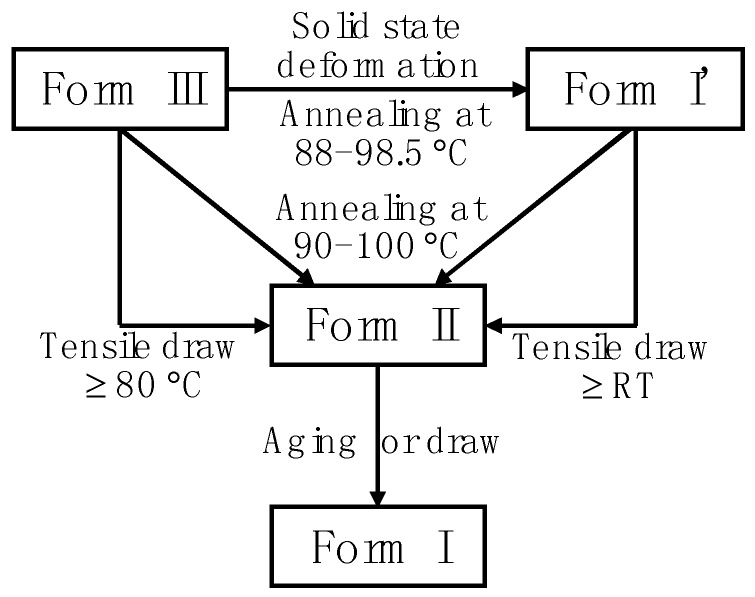
A sketch shows the phase transitions between different crystal forms of iPBu under varied conditions.

As mentioned in the Introduction, the stability of various crystals for a given polymer is different. This leads to the transition between different phases, either spontaneous or factitious, under suitable condition is an inevitable phenomenon. The iPBu exists complicated phase transition behavior. As presented in Figure 20, form III can transform into I’ transition during solid-state deformation or into form II simply up heating as well as through tensile draw at temperatures above 80 °C [122]. Form I’ can transform into form II through tensile draw at room temperature or thermal annealing as well [123]. Form II will always transform spontaneously into form I since it is unstable under ordinary conditions [26,89]. This has stimulated extensive studies on the phase transition behavior of iPBu [83,124,125,126,127,128,129,130,131,132,133,134,135,136,137,138,139,140,141,142,143,144,145,146,147,148]. The related works focus mainly on the mechanism of the phase transition and the transition kinetics as well as its affecting parameters. In this section, we describe the recent progress made in these fields.

### 4.1. Phase Transition Mechanism of iPBu

**Figure 21:**
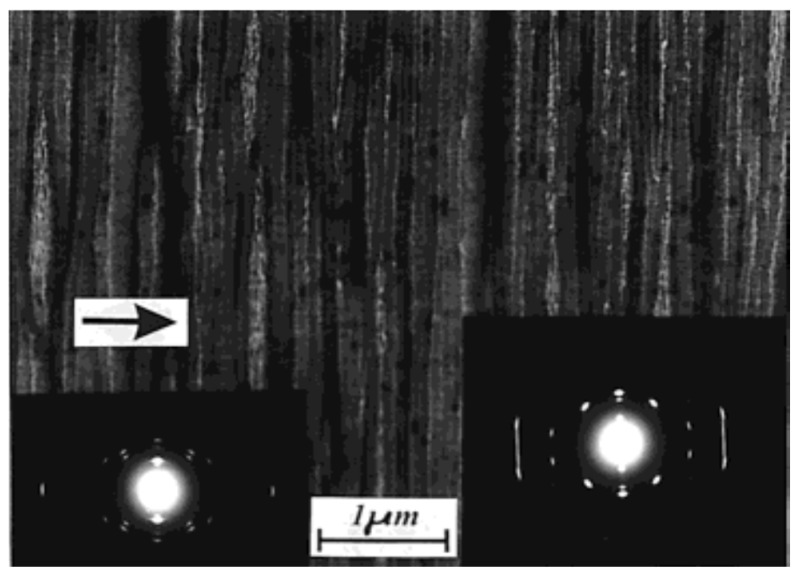
BF electron micrograph and its corresponding electron diffraction patterns (insets) of an oriented iPBu thin film taken during the II-I phase transition. The electron diffraction patterns in the lower left and lower right corners correspond to the form II and form I crystals, respectively. Reproduced with permission from Ref. [90], copyright 2003 American Chemical Society.

**Figure 22:**
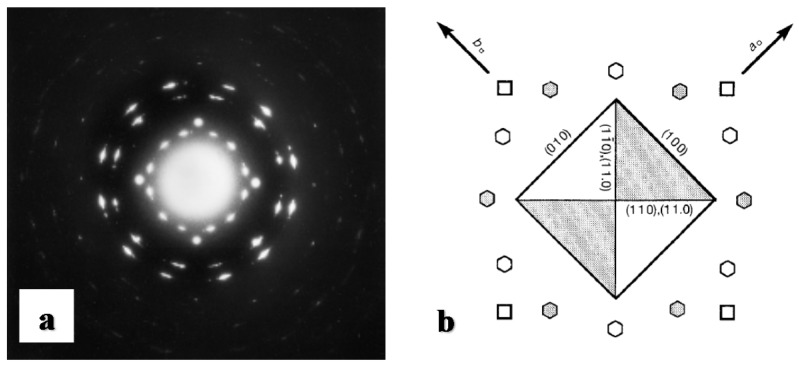
(**a**) An electron diffraction pattern taken during the phase transition process of iPBu from form II to form I. Note the twelve reflection spots of transformed form I superimposed on the original form II diffraction pattern, displaying its twinned structure. Reproduced with permission from Ref. [33], copyright 2002 Wiley Periodicals, Inc. (**b**) A sketch showing the origin of twinned crystal lattice orientations of the transformed form I iPBu according to Holland and Miller [29]. The growth sectors of original form II crystal are shown in light and shaded triangles. The surrounding squares and hexagons represent the diffraction spots contributed by the original form II and transformed form I crystals, respectively. Reproduced with permission from Ref. [134], copyright 1994 Chapman & Hall.

The irreversible spontaneous solid phase transition from form II to form I of iPBu was first reported by Natta et al. in the 1960 [124]. In the more than past six decades, the study on the iPBu phase transition behavior has be well continued up to date. Studies on the II-I phase transformation of uniaxially oriented thin films with edge-on lamellar structures showed that the original form II and the transformed form I exhibits the same molecular chain direction, see Figure 21. Owing to the fiber orientation the highly oriented thin film, no other crystallographic relationship was found between the forms II and I crystals. Studies on the flat-on illustrated a “twinned” orientation of the transformed form I crystals, as displayed in Figure 3, which differs from that of the directly formed I’. The slow solid phase II-I transition makes it possible for following the transformation process by electron and X-ray diffractions. Figure 22a shows an electron diffraction pattern of iPBu taken during the phase transition, i.e., before the complete transformation of the form II crystals [33]. It contains one diffraction set of the original form II and two sets of the twinned form I crystals. A careful analysis of the superimposed electron diffraction patterns indicates that the original form II and transformed form I exhibit common (110) lattice planes. An explanation of this twinned structure was then first proposed by Holland and Miller [29]. They related the twinned orientation of transformed form I to the existence of growth sectors of the parent crystals. As schematically presented in Figure 22b, it was suggested that the light and shaded sectors, which exhibit different folding directions, resulted in the appearance of the light and shaded diffraction spots of the transformed form I, respectively. 

**Figure 23:**
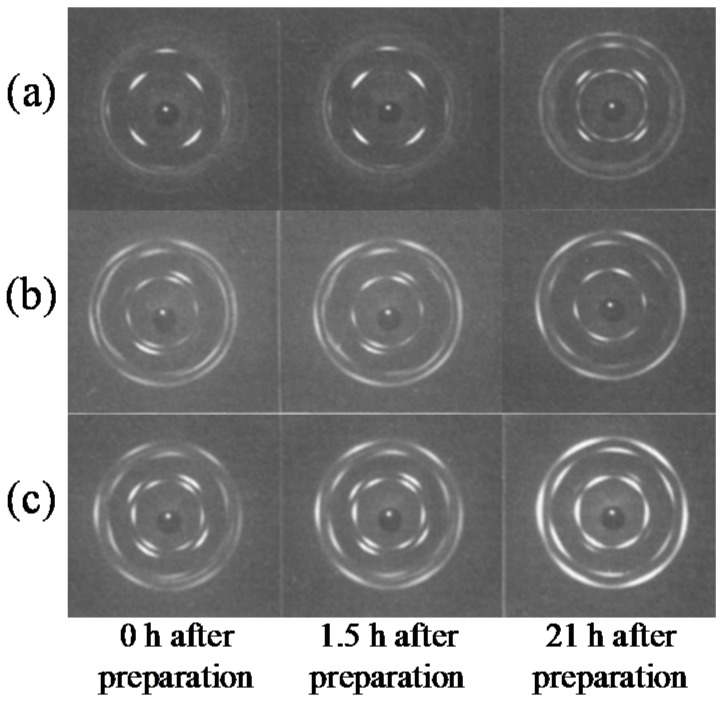
X-ray diffraction patterns of: non-sheared (**a**); sheared parallel (**b**); and perpendicular (**c**) to the [110] direction of the oriented iPBu samples taken during the II-I phase transition for 0, 1.5 and 21 h after sample preparation. Reproduced with permission from Ref. [130], copyright 1985 Springer-Verlag.

**Figure 24:**
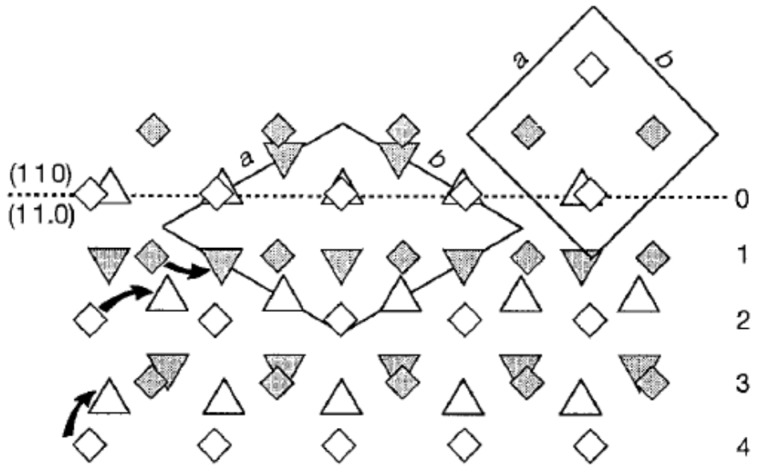
Scheme of the transformation mechanism of iPBu from form II to form I. A tetragonal cell of form II and a trigonal (hexagonal) cell of form I are indicated. The 11/3 and 3/1 helices of forms II and I are represented as squares and triangles, respectively. The different helical hands are shown as light and shaded. The transformation is initiated in layer 0 and propagates both sideways and downwards. The lateral shifts of chains in layers 1 and 2 and significant lattice shrinkage in the vertical direction is manifested by helix movements in layer 4. Reproduced with permission from Ref. [134], copyright 1994 Chapman & Hall.

The above explanation was quickly invalidated when two orientations of form I within a single growth sector of form II iPBu, and only one form I crystal orientation was created in different sectors of the original form II crystal, were observed [29,134,136]. In addition, the results of the shear-induced phase transformation of oriented iPBu flat-on crystals, which were melt-crystallized in a temperature gradient [130], do not support the transformation model proposed by Holland and Miller. As presented in Figure 23, it was found that twinned orientations of form I iPBu crystals were created after complete transformation of the non-sheared sample with from II crystals (Figure 23a). However, if the sample was sheared either parallel (Figure 23b) or perpendicular (Figure 23c) to the [110] direction of the oriented iPBu sample, except for an accelerated transformation rate, only six-point X-ray patterns were observed. Those six-point X-ray patterns were rotated at right angle to each other. Based on all these experimental results, a rational explanation regarding the form II to form I phase transition of iPBu has been proposed with full consideration of conformational and steric constraints [130,134,135]. The structural aspect of the transformation is schematically presented in Figure 24. Taking the packing of 11/3 helices with alternation of helical hands along both *a*- and *b-*axes of form II into account, the (110) layers contain isochiral helices, with successive layers being antichiral. The trigonal unit cell of form I with 3/1 helices has also the antichiral alternation of (110) layers made of isochiral helices. In this case, the parallel alignment of the (110) planes of both forms II and I iPBu crystals reveals the constraints set by preservation of the helical hands [130,135]. The transformation is thus made with minimum helix displacement and no energetically unrealistic change of helix chirality.

**Figure 25:**
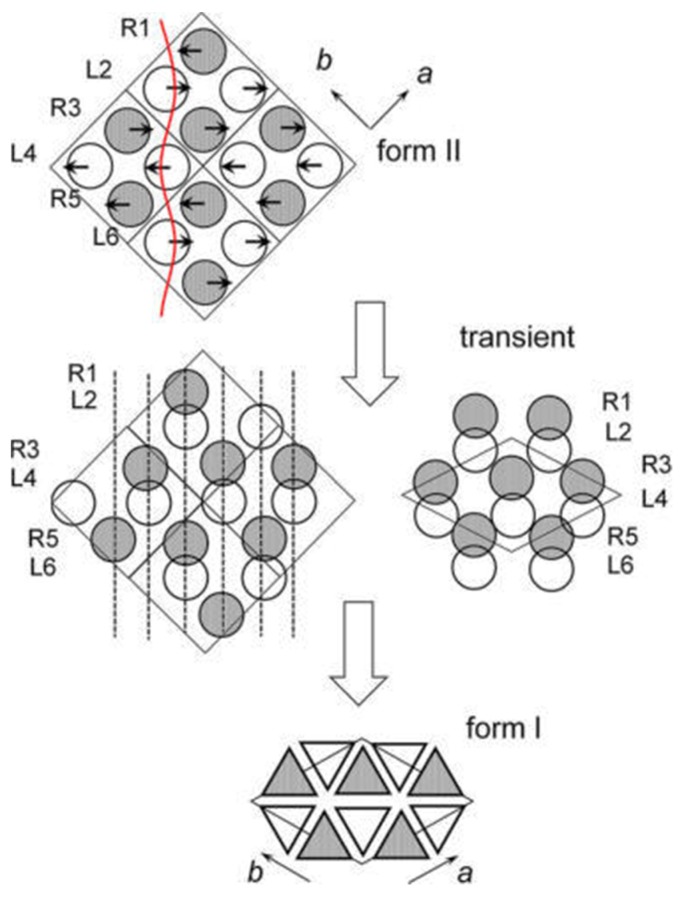
A possible phase transition model of iPBu crystal from form II to form I proposed by Tashiro et al. The R and L chains move to the mutually opposite direction with the phase angle π along the [110] direction. This translational lattice mode results in the formation of a transient structure, which transforms to the chain packing mode of form I. The change in chain conformation is ignored here, which should occur cooperatively in parallel to the translational mode shown here. Reproduced with permission from Ref. [23], copyright 2016 American Chemical Society.

Moreover, the solid phase transition may be realized cooperatively or non-cooperatively [89]. In the cooperative process, the molecular stems change from one crystal structure to the other by a coordinated movement and the relative position of nearest neighbors remain nearly unchanged during the transformation. On the other hand, in the non-cooperative transformation, the molecular stems moving into the new crystal form are not necessarily the same as that after transition. For iPBu, a very recent study by Tashiro and co-workers [23] suggests that the molecular chains of right (R) and left (L) handed iPBu helices move cooperatively during transition with a definite phase relation. It is assumed that the R and L chains in the form I crystal make a pair. These pairs are arrayed in a hexagonal packing mode and orient along the [110] direction. On the other hand, the R and L chains in the form II crystal are arranged in a zigzag mode along the [110] direction. The idea of Tashiro et al. is presented in Figure 25. They assumed that the pairs of R and L helixes similar to that of form I can be simply produced by the opposite movement of the R and L helixes. For example, the R1-L1 pair is caused by movement of R1 and L2 chains toward left and right directions, respectively. In the same way, the R3 and L4 chains move to the mutually opposite directions to make another pair. These pairs will arrange first in a transient similar to that observed for the crystal form I, and finally transform to the energetically stable chain packing mode of form I. The formation of a transient structure has also been reported by Li and coworkers [149].

**Figure 26:**
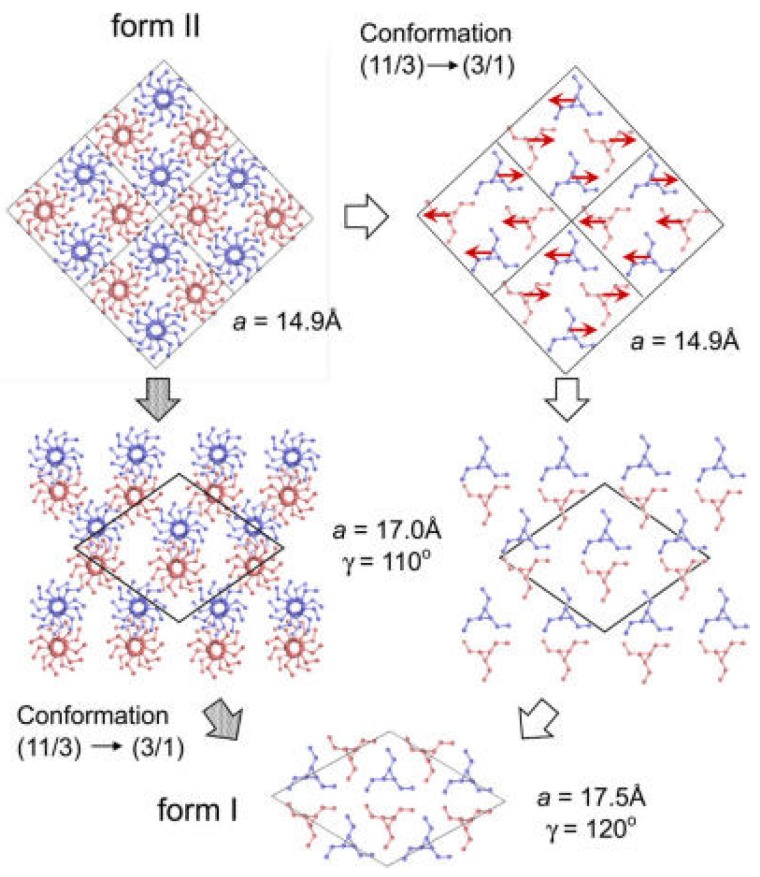
Schematic representation showing the possible chain conformation change of iPBu during form II to form I transformation. Reproduced with permission from Ref. [23], copyright 2016 American Chemical Society.

The above discussion has ignored the change in chain conformation. It happens, however, during transition from 11/3 to 3/1 helixes. As illustrated in Figure 26, there may be two possibilities regarding to the conformational change. The first one is that the R and L 11/3 chains change to the 3/1 helixes before pairing. The other one is that the 11/3 to 3/1 conformation change occurs in the pair of the R and L 11/3 chains. In the actual transition, the conformational change and the translational motion of the chains may take place cooperatively through the lowest energy path from form II to form I.

### 4.2. Phase Transition Kinetics of iPBu

**Figure 27:**
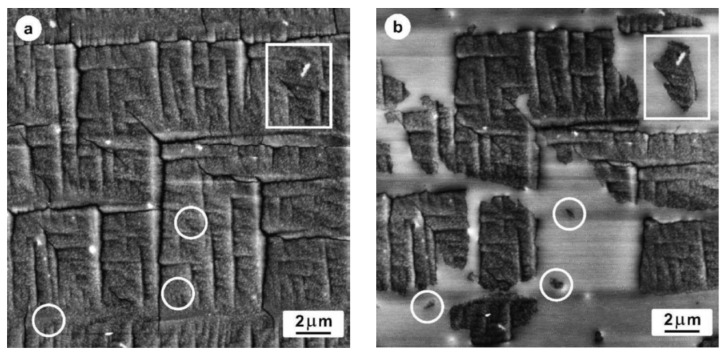
AFM height images of the iPBu flat-on crystals scanned at (**a**) room temperature and (**b**) at 124 °C. The used sample was initially crystallized at 100 °C for 24 h and then aged at room temperature for five days. The white markers indicate the same area of the sample. Reproduced with permission from Ref. [150], copyright 2004 Elsevier Ltd. All rights reserved.

The exact origin at a molecular level and driving force that initiate the spontaneous form II to form I transition is not quite clear yet. Nevertheless, the increased nucleation density of bulk crystallized iPBu samples under shear as well as at high pressure suggests that the solid-phase transition is most likely induced by mechanical or thermal (caused by temperature change) stresses [20,89,132,134]. Our morphological study on the iPBu during transition process found that a spontaneous transformation of all the lamellae in the spherulites with edge-on lamellae [150]. This indicates that the nucleation of form I is sporadic in time and place, in agreement with the literature [89,132]. The morphological feature of transformed form I provides more information about phase transition. Figure 26 shows two AFM height images of the iPBu flat-on crystals crystallized at 100 °C for 24 h and aged at room temperature for five days, which were scanned at room temperature and at 124 °C, respectively. The white markers indicate the same area of the sample. In Figure 27b, the untransformed form II iPBu crystals melt, while form I remain unchanged owing to a higher melting point (ca. 136 °C). One can see that the nucleation of the stable form I crystals starts most likely at crystalline side surfaces or corners of the flat-on form II crystals. This may serve as an indirect evidence for the stress induced solid-phase transition in the following way. In the boundary region of the flat-on crystals, the long iPBu molecular chains packed in different crystals may be strengthened. Stresses exerted by these taut tie molecules may provide the nucleation sites of the stable form I. 

Studies on the transformation kinetics showed that the form II to form I phase transition of iPBu obeys an exponential law and therefore belongs to a first order process, including nucleation and crystal growth. There is, however, conflict about the rate-determining step of the transition. Gotdbach [147,148] and Gohil et al. [89] claimed that the nucleation of form I iPBu crystal is slow and is therefore the controlling step of the phase transition. The experimental support of this is that some form II monolamellar iPBu crystals in thin film grown from a polymer solution can remain unchanged after one month. In addition, the single orientation of the transformed form I indicates a comparatively rapid propagation of the transformation within the whole crystalline lamella after the nucleation event [134]. The result shown in Figure 27 also supports the conclusion that the nucleation of the form I crystals is the rate-limiting step of the form II to I conversion. It is clear that the iPBu lathlike microcrystallites are either in crystalline or in molten states at 124 °C. This infers that each nucleus initiates the transition of the completely separated crystalline piece. As long as the nucleus is generated, the transformation of an isolated crystal can be finished quickly in a certain time. On the other hand, Chau and Geil [132] indicated profuse, instantaneous nucleation at early stages of the transformation, followed by a second process of random nucleation. They have therefore concluded that the growth of the stable form I is the rate-determining step. The above-mentioned discrepancy is actually associated to the different experimental conditions used by different groups. 

**Figure 28:**
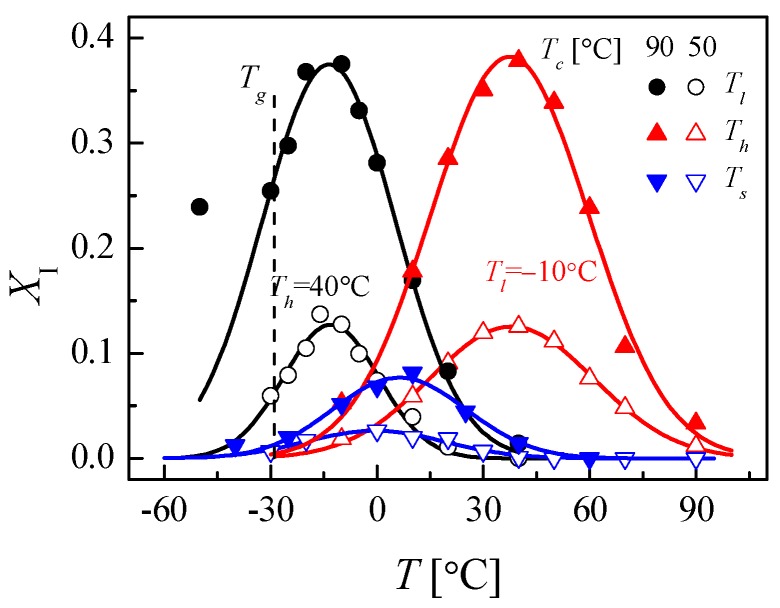
Form I crystal fraction vs. temperature profiles of iPBu samples crystallized isothermally at 50 and 90 °C, respectively. The black curves were obtained by annealing first at different temperatures (*T_l_*) for 60 min and then at 40 °C for 220 min. The red curves were obtained by annealing first at −10 °C for 60 min and then at different temperatures (*T_h_*) for 220 min. The blue curves present data of samples simply annealed at different temperatures for 280 min. It is clear that, for both samples, the optimal temperatures for the nucleation or growth of form I crystals during II-I transition are around −10 and 40 °C, respectively. However, a synergistic effect of nucleation and growth makes the phase transition at a fixed single temperature most effectively at room temperature. Reproduced with permission from Ref. [143], copyright 2016 American Chemical Society.

Recent studies on the nucleation and growth of form II to I polymorphic transition of iPBu through stepwise annealing indicated the existence of favored temperature for the nucleation and growth of the form II to form I transition, respectively [143,146]. As presented in Figure 28, it was found that the temperature corresponding to a maximum transformation rate at the initial stage is approximately −10 °C, whereas the temperature for a quick transformation at the later stage is around 40 °C. The quick transformation at low temperature in the early stage was related to a faster nucleation. As long as the form I nuclei formed, a faster propagation of the crystals is naturally expected at higher temperatures. In conclusion, the optimal temperatures for the nucleation or growth of form I crystals during II-I transition are −10 and 40 °C, respectively. Therefore, by annealing first at −10 °C for short time to induce the nuclei and then at 40 °C to promote the form I crystal growth provides an ideal pathway for accelerating the solid-phase of iPBu.

### 4.3. Factors Influencing the Phase Transition of iPBu

Many factors except for the temperature at which the transformation performed, such as external or thermal stress, high pressure, pressured CO_2_, crystallization temperature, molecular characteristics, cross-linking, sample thickness and so on, influence the phase transition of iPBu [12,30,141,151,152,153,154,155,156]. The external or thermal stress and high pressure tremendously accelerate the phase transition [30,142,153,154,155,156,157]. This is acceptable since stresses are considered as the cause of the phase transition. The fast phase transition of iPBu I in pressured CO_2_ is explained in terms of the diffusion of CO_2_ into polymer, which enhances the chain or chain segments mobility [141,151,156]. It is rational since the high chain mobility favors the conformational adjustment from 11/3 to 3/1 helixes on the one hand and the related translational motion of the molecular chains during form II to form I transition on the other hand. The effect of crystallization temperature on the II-I phase transition behavior of the iPBu is well illustrated in Figure 28. An overall increase of form I fraction for the sample crystallized isothermally at 90 °C can be observed in all three cases. The enlarged difference between crystallization and transformation temperatures is thought to increase of driving force for overcoming the energetic barrier for nucleation and then accelerate the overall phase transition. 

The influence of molecular characteristics includes molecular weight and incorporation of co-units (i.e., copolymers). It was confirmed that the transformation rate can be tailored by incorporation of random 1-alkene co-units in the butene-1 chain. While the co-units with short-chain olefins, such as ethylene or propylene, accelerates the Form II to I transformation rate, the II-I transition is retarded for copolymers containing linear 1-alkenes with more than five carbon atoms [146,152,157,158,159]. Moreover, it was found that the incorporation of ethylene co-units into isotactic poly(butene-1) also affects the optimal temperatures of the maximum transformation rate [146]. The influence of co-units on the transformation rate has been ascribed to the varied composition of the rigid amorphous portions at the lamellar basal planes of the crystals. 

**Figure 29:**
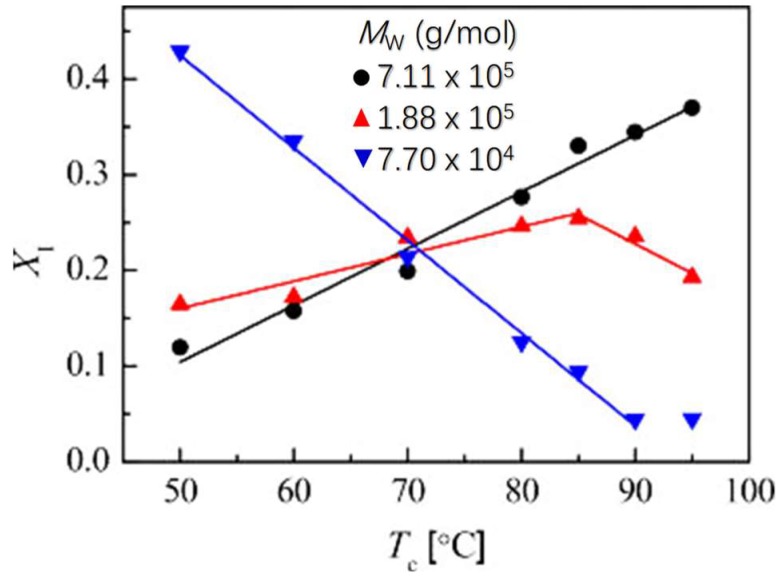
Form I crystal fraction vs. crystallization temperature profiles of iPBu samples with different molecular weight as indicated. The samples were prepared by melting at 160 °C for 8 min to erase the thermal history and then crystallized at different temperatures for the complete the crystallization of form II. The thus prepared sample were annealed at −10 °C for a 60 min nucleation followed by annealing at 40 °C for a 220 min growth of form I crystals. The contents of forms II and I were obtained via melting peak fitting and integrating procedure considering exponentially modified Gaussian functions. Reproduced with permission from Ref. [160], copyright 2017 American Chemical Society.

For the molecular weight influence on the phase transition, Chau and coworkers [132] reported that the transformation rate is lower in the high molecular weight sample. Based on this result they concluded that taut tie molecules are not essential for phase transformation to occur. They further suggested that chain ends, which increase with decreasing molecular weight, play a role in the phase transition. Men et al. have also followed the phase transition behavior of three samples with different molecular weight under the same crystallization and phase transition conditions [160]. As displayed in Figure 29, at relatively low crystallization temperatures, e.g., lower than 60 °C, the sample with low molecular weight transforms indeed faster than the high molecular weight one. However, the dependence of transition rate on crystallization temperature is quite different for the high and low molecular weight samples. The transition rate of the high molecular weight sample increases clearly elevated crystallization temperature of the initial form II crystals, while a negative correlation between transition rate and crystallization temperature was obtained for the low molecular weight sample. Consequently, the content of the transformed form I crystals of the low molecular weight sample crystallized at temperature above 80 °C is much small compared with that of the high molecular weight one. They correlated the different behavior to the intercrystalline links. It was speculated that the long spacing of high molecular weight sample is smaller than the radius of gyration of the chains in the melt. As a result, an abundance of taut tie molecules connecting the crystalline lamellae can be formed during crystallization, which produce higher internal stress during cooling from crystallization temperature to room temperature. On the other hand, the intercrystalline links in low molecular weight sample are expected to decrease with increasing lamellar thickness. Consequently, the phase transition of it slows down with elevated crystallization temperature.

The effect of cross-linking on the phase transition is tested by electron irradiation the sample of form II iPBu crystals [128]. The experimental results demonstrate that irradiation slows down the rate of II-I transition. The suppression degree of the phase transition is directly dependent on the irradiation dose. This implies that radiation-induced crosslinking suppressed the normal expansion of the helix and may be also the translational movement of the molecular chain segments. This has been further confirmed by comparing the transition rate of the sample irradiated in air and vacuum environment. It was found that the sample irradiated in air transforms faster than in vacuum. This has been related to the scavenge of radiation-produced radicals by oxygen, which prevents the intermolecular crosslinking of the iPBu. It has also been found that remolding an irradiated sample of form II iPBu causes an immediate transformation into form I rather than form II as usually observed for the non-irradiated samples. This has been explained in terms that the crosslinks formed by irradiation were excluded from the crystalline lamellae, leading to the formation of thinner and less perfect crystallites which convert rapidly to the stable form I crystals.

The rate of form II to form I phase transition is found to be also sample thickness dependent [127,128,132,161]. It was first found by Boor Jr. and Mitchell [159] and quickly confirmed by Clampitt et al. [127] and Luongo et al. [128]. Clampitt et al. reported that the thinner the film the faster the II-I conversion rate. To find a reasonable explanation the thickness-dependent phase transition, Luongo and Salovey studied the transition rate of the thin and thick samples by attenuated total reflectance (ATR) infrared technique, which reflects the same depth of the surface structure of the samples. They found that the rate of II-I transformation is actually equal and both rapid on the surfaces of both thin and thick films. It is therefore believed that the more rapid cooling of the surfaces during the sample preparation induces strains on the film surfaces analogous to stretching, which promotes the rapid phase transition of the surface layer.

## 5. Conclusions and Perspectives

IPBu is a polymer with pronounced polymorph and complicated phase transition behavior. Its thermodynamically most stable form I is a thermoplastic material of industrial interest due to its excellent physical and mechanical properties such as high thermal stability, hardness, stiffness, and strength. Unfortunately, form I iPBu is hardly produced since it goes from melt quickly into its kinetically favored metastable form II. Due to its lower stability, the form II iPBu crystals transform spontaneously into form I at room temperature storage. Even though the solid-phase transition endows the aforementioned high-performance of the material, it limits considerably the applications of iPBu since the transformation results in volume shrinkage and shape deformation of the products due to the difference structures of forms II and I. Therefore, from a practical standpoint, bypassing the unstable form II crystallization is of great significance. This leads to a substantial amount of work on seeking straightforward ways for a direct crystallization of iPBu into stable form I, particularly under processing conditions. Meanwhile, pathways for accelerating the form II to I transition becomes another focus of research before a right method of direct formation of form I has been found. After nearly 70 years of unremitting efforts, great progress has been made in several aspects including crystal structures and their selection roles during crystallization, phase transition mechanism and kinetics, and the possible influencing factors for governing the crystallization and phase transition processes.

First, refinement of the crystal structures of different forms has been achieved recently. For example, in the form I crystal structure, the right- and left-handed helical chains form pairs and pack together around the three-fold rotation axis in a way that the right-handed helixes are surrounded by the left-handed helixes and vice versa. Two possible space groups, i.e., the *R*3*c* or $R\bar{3}c$, can be adopted for this structure feature. Based on quantitative analysis of the two-dimensional X-ray diffraction data, an $R\bar{3}c$ space group was suggested in 1997. However, the recent study has further refined the crystal structure of form I as a $P\bar{3}$ space group. In addition, form II of iPBu has a tetragonal unit cell packed by 11/3 helix conformation with opposite chirality and a repeating period along the chain axis of 21 Å. It has been determined as a $P\bar{4}b2$ space group recently. This leads to an alignment of the right- and left-handed helical chains in the unit cell positioned alternately with the statistical disorderliness about the upward and downward directionality.

Second, in the field of crystal structure modulation of iPBu, several methods have been established for the direct formation of iPBu materials with form I crystal structure. From the synthetic point of view, both incorporation other α-olefin co-units and introduction of stereo-defects into the molecular chains provide an efficient way for controlling the crystallization of iPBu in desired forms. From the point of processing, a striking example is the crystallization of iPBu under depressed CO_2_, which favors the formation of iPBu crystals with form I structure. Another way for a direct production of form I iPBu is to control the melting status of the material and the subsequent crystallization condition. In this way, the interplay between the domain size of aligned chain segregates previously included in the crystal lattice and the size and energy barrier of the critical nucleus corresponding to different crystalline forms play an important role for the polymorphic selection.

Third, new form II to form I transformation mechanism has been proposed according to the refined crystal structures of both forms I and II. It is now believed that pairs of R and L helixes similar to that of form I can be simply produced by the opposite movement of the R and L helixes in form II. These pairs will arrange first in a transient state similar to that observed for the crystal form I, and finally transform to the energetically stable chain packing mode of form I. As for the change in chain conformation during phase transition, two possibilities have been speculated. The first one concerns the change of the R and L chains from 11/3 to 3/1 helixes before pairing, while the other one suggests the 11/3 to 3/1 conformation change occurring in the pair of the R and L 11/3 chains. Actually, the conformational change and the translational motion of the chains may take place cooperatively through the lowest energy path from form II to form I in the actual transition. 

Fourth, for the form II to form I phase transformation kinetics, a first order transition including nucleation and growth is established. Recent progress in this aspect rests on the separation of temperature effects on the nucleation and growth processes, respectively. It is well documented that the nucleation of form I crystals takes place most efficiently at −10 °C, while the optimal temperature for form I crystal growth is around 40 °C. In this way, by annealing the initial form II crystals first at −10 °C for short time to induce the nuclei and then at 40 °C to promote the form I crystal growth provides an ideal pathway for accelerating the solid-phase of iPBu. Moreover, it is found that all of the factors affecting the crystallization of iPBu in different forms influence the form II to form I solid phase transition as well.

Fifth, considering that the exact origin at a molecular level and driving force that initiate the spontaneous form II to form I transition is not yet clear, further studies on the physics of the transformation is highly desirable in connection with advanced applications of iPBu. It is also sufficient interest to warrant continuous research with sophisticated techniques. For this aspect, molecular dynamics calculation and simulation may play a greater role for a fully understanding of it at molecular level.

## Figures and Tables

**Table 1:** The epitaxial relationship of iPBu on different substrates in different phases [117,118].

Substrate	Crystalline phase	Number of orientation	Epitaxial orientation relationships
2-Quinoxalinol	III	1	(110)_iPBu_ // (001)_2-Qol_ C_iPBu_ // [010]_2-Qol_
Benzoic acid	II	2 (∠94°)	(100) or (010)_iPBu_ // (011)_BA_ C_iPBu_ // [110] or [$\bar{1}10]$_BA_
4-Bromobenzoic acid	II (+)	2 (∠50°)	(100) or (010)_iPBu_ // (100)_4BrBA_ C_iPBu_ // [032] or [03$\bar{2}$]_4BrBA_
	I’ (−)	2 (∠22°)	(110)_iPBu_ // (100)_4BrBA_ C_iPBu_˄ [001]_4BrBA_ = ±11°
4-Chlorobenzoic acid	I’ (+)	1 or 2 (∠22°)	(110)_iPBu_ // (100)_4ClBA_ C_iPBu_˄ [001]_4ClBA_ = ±11°
	II (−)	1	(100)_iPBu_ // (100)_4ClBA_ C_iPBu_ // [032]_4ClBA_
Potassium hydrogen 4-chlorobenzoate	I’	2 (∠60°)	(110)_iPBu_ // (100)_PH4ClB_ C_iPBu_˄ [001]_PH4ClB_ = ±30°
Potassium 4-chlorobenzoate	I’	2 (∠60°)	(110)_iPBu_ // (100)_P4ClB_ C_iPBu_˄ [001]_P4ClB_ = ±30°
Potassium 4-bromobenzoate	I’	2 (∠60°)	(110)_iPBu_ // (100)_P4BrB_ C_iPBu_˄ [001]_P4BrB_ = ±30°

(+) means the majority phase, (−) means the minority phase, ∠X° in the brackets describes the angle between iPBu molecular chains of the observed two orientations.
